# Critical Evaluation of Laboratory Potentiometric Electronic Tongues for Pharmaceutical Analysis—An Overview

**DOI:** 10.3390/s19245376

**Published:** 2019-12-05

**Authors:** Małgorzata Łabańska, Patrycja Ciosek-Skibińska, Wojciech Wróblewski

**Affiliations:** 1 Plant Breeding and Acclimatization Institute—National Research Institute, Bonin Research Centre, Bonin 3, 76-009 Bonin, Poland; 2 Chair of Medical Biotechnology, Warsaw University of Technology, Noakowskiego 3, 00-664 Warsaw, Poland; pciosek@ch.pw.edu.pl (P.C.-S.); wuwu@ch.pw.edu.pl (W.W.)

**Keywords:** sensor arrays, ion-selective electrodes (ISEs), pharmaceutical analysis, dissolution studies, taste-masking

## Abstract

Electronic tongue systems equipped with cross-sensitive potentiometric sensors have been applied to pharmaceutical analysis, due to the possibility of various applications and developing new formulations. Many studies already proved the complementarity between the electronic tongue and classical analysis such as dissolution tests indicated by Pharmacopeias. However, as a new approach to study pharmaceuticals, electronic tongues lack strict testing protocols and specification limits; therefore, their results can be improperly interpreted and inconsistent with the reference studies. Therefore, all aspects of the development, measurement conditions, data analysis, and interpretation of electronic tongue results were discussed in this overview. The critical evaluation of the effectiveness and reliability of constructed devices may be helpful for a better understanding of electronic tongue systems development and for providing strict testing protocols.

## 1. Introduction

Growing awareness and requirements of consumers and manufacturers lead to increased and more advanced quality control of raw materials, final products, and entire production processes. Well-known instrumental methods such as chromatography or spectroscopy are routinely employed for quality control in laboratories worldwide. Despite their numerous advantages, they require sample pre-treatment and heavy laboratory facilities, which involve high maintenance and operation costs. Therefore, there is a burning need to develop modern analytical tools for reliable, rapid, and inexpensive analysis of multicomponent samples. One potential approach to this challenge is a multisensory device, referred to as electronic tongue, which is dedicated to automatic qualitative and quantitative analysis of complex liquid samples and to recognition of their characteristic properties [1].

According to IUPAC (International Union of Pure and Applied Chemistry) definition, an electronic tongue (ET) is a device combining an array of low-selective sensors and advanced mathematic procedures used for signal processing based on the pattern recognition (PARC) and/or multivariate data analysis methods such as PCA (principal component analysis) or ANNs (artificial neural networks) [2]. The term ’electronic tongue‘ refers to the similarity to the human gustatory system and for the first time was used in 1996 [3], although the first attempt to implement operating principles of highly developed biological senses to an artificial sensory system had been described in early 1960s [4]. The first work which can be regarded as dealing with a multisensory approach for odor analysis was published in 1982 by Persaud and Dodd [5]. Since then, the concept of “electronic nose” has been intensively investigated in terms of sensor arrays [6,7] and pattern recognition methods [8,9]. As a result, many electronic nose systems for the recognition of gas mixtures and/or identification of gas individuals are commercially available [10]. In 1985, Otto and Thomas proposed the first system for the analysis of liquid samples based on a sensor array [11]. For over 30 years several research groups have focused on the development and application of such devices. The evolution of artificial senses within the last decades was summarized in various reviews [1,12,13,14,15].

A wide variety of chemical sensors is applied in the design of the electronic tongue systems. Potentiometric sensors were the first utilized in such systems [16] and still remain the most widely used [17], especially in commercially available systems [18]. Over time, other chemical sensors like voltammetric [19], optical [20], piezoelectric [21], and impedimetric [22] ones were exploited as well. The essence of electronic tongue analysis lies in an overall assessment of complex samples, allowing to distinguish and classify them as well as estimate the content of their specific components [23]. Patterns of sensor array responses form the characteristic chemical images/fingerprints of samples. However, the large number of features describing a chemical image of the sample demands methods for the extraction of significant features from sample classes. To this aim, multivariate data analysis by means of chemometric tools has been successfully applied [24]. Therefore, various pattern recognition techniques are employed for data analysis of electronic tongue measurements. The most commonly used are: PCA [25,26], partial least squares (PLS) [27], support vector machine (SVM) [28], and ANNs [29].

So far, numerous publications have demonstrated a wide range of applications of electronic tongues. Extensive surveys on the usage of those systems to food and beverage analysis [30,31], bioprocess control [32], environmental monitoring [33], and medicinal diagnosis [34] were published. Moreover, more recent reports consider the use of electronic tongues systems for pharmaceutical analysis, including the evaluation of taste-masking efficiency, dissolution tests, as well as elaboration and quality control of drugs, herbals, and medicinal plants (e.g., determination of batch-to-batch uniformity, characterization of active pharmaceutical ingredients) [35,36].

Taste is one of the primary factors affecting the drug adherence, especially for pediatric patients. Moreover, changes in the European regulatory requirements [37] initiated the development of medicines intended for pediatric use and require the development of age-appropriate formulations. Since the majority of active pharmaceutical ingredients (APIs) exhibit bitter taste, various taste-masking approaches have been developed in order to reduce unpleasant sensation [38,39,40]. The efficiency of taste-masking, obtained by addition of sweeteners [41], microencapsulation [42], hot-melt extrusion [43], cations exchangers, and complexing agents [44] has been assessed with commercially available models and laboratory prototypes of electronic tongues. In all cases, the utilized devices were able to distinguish samples of different taste profiles. However, it must be underlined that these systems are based on chemical sensors, which are not able to sense taste itself and they do not reproduce human sense of taste. Therefore, the obtained correlations between sensor array responses and taste sensations are based only on discrimination between samples of different compositions.

Pharmaceutical technologies which are constantly being developed provide new formulations and dosage forms demanding a comprehensive analysis. Researchers working on the development of pharmaceutical formulations consider the use of artificial sensing systems as a supplementation to conventionally used methods. Recently, a commercial taste sensing system (TS-5000Z) was used to differentiate novel oral dosage forms (i.e., orally disintegrating tablets (ODTs)) containing diclofenac [45] and lyophilisates containing cetirizine hydrochloride [46]. A laboratory prototype of an electronic tongue was able to distinguish microparticles, lyophilisates, and ODTs containing dihydrochloride cetirizine [47]. Taste-masked ODTs and self-emulsifying drug delivery systems (SEDDS) with cyclosporine were evaluated using the αAstree electronic tongue. The device discriminated ODT and SEDDS samples containing API from pure API [48]. Another example of ET application is improving formulation development. Discrimination of taste attributes of three diclofenac drug forms and distinction between differently designed formulations were achieved [49]. Moreover, the αAstree electronic tongue was used to compare the palatability of the original and eight generic versions of famotidine ODTs. Euclidean distances on PCA plot between chemical images of products from different brands and the original product were correlated with bitterness intensities obtained by the gustatory sensation test. High correlation (R^2^ = 0.986) was obtained [50]. 

Electronic tongue systems are gaining more attention as a tool for dissolution studies. Recently, the potentiometric multisensory system (including 24 sensors based on PVC-plasticized membranes and standard pH glass electrode) was applied to perform off-line and in-line measurements. During off-line measurements, the samples were withdrawn from dissolution vessel by a sampling system at specific time points of the dissolution process. In the case of the in-line electronic tongue, the sensor array was placed into the dissolution vessel and the electrode signals were continuously recorded every eight seconds. It was pointed out that both off-line and in-line electronic tongue results were comparable to dissolution studies performed by in-line UV measurements (i.e., UV probe immersed in the dissolution vessel). Although a certain inconsistency in dissolution profiles obtained by two different methods during in-line measurements was noticed [51]. Studies on the evaluation of complexation potential of brompheniramine maleate and tannic acid for sustained release and taste masking effects was conducted using the αAstree electronic tongue [52]. Electronic tongue discriminated samples with various drug to excipient ratio, allowing to optimize such ratio in the pharmacological product.

Furthermore, ETs are applied for the analysis of herbal medicines. An in-house fabricated multichannel sensor, incorporating an array of artificial lipidpolymer membranes, recognized extracts from different parts of plant, age, batch-to-batch variation, and mode of extraction of *Eurycoma longifolia* [53]. Various taste-masking techniques, measurement protocols, analytical procedures, as well as comparisons between the performances of commercially available systems applied to pharmaceutical analysis were reported [54,55,56]. Finally, the reliability of six different electronic tongues (commercial and laboratory prototype systems) applied to the same set of pharmaceutical samples were evaluated [57].

For more than 15 years our studies were focused on developing an electronic tongue for the recognition of various types of food, environmental, bioprocess, and biological samples. The aim of this paper is to evaluate the effectiveness and reliability of electronic tongue systems based on potentiometric sensors for pharmaceutical analysis. Various architectures of sensor arrays, experimental strategies, data processing techniques, as well as reference methods applied in our recent works were discussed, leading to important conclusions useful for further studies.

## 2. Different Architectures of Sensor Arrays

A sensor array is the primary element of an electronic tongue. Most models are based on cross-selective (so called non-specific) and cross-sensitive chemical sensors. Even though they are not specific singularly, an array of sensors can provide an unique profile (fingerprint) for each sample, based on the concentration of the different chemical species dissolved in it. Thanks to this feature, sensor arrays are able to provide much more information about samples and more comprehensive chemical images of them. Due to numerous advantages (e.g., well-known principle of operation, low cost, simple set-up, possibility of miniaturization), potentiometric sensors are definitely the most popular tools for the fabrication of commercially available systems and laboratory prototypes of electronic tongues. The principle of operation of a potentiometric sensor is based on the measurement of its potential against a reference electrode, referred to as electromotive force (EMF). The electrical potential is formed across the phase boundary between the sample and the ion-selective membrane—the key element of potentiometric sensors. The composition of the membrane determines the sensor selectivity (i.e., the influence of the activity of various ions present in the sample on the phase boundary potential) via selective binding processes occurring at the membrane–solution interface (e.g., ion-exchange or complex formation, in the case of ion-selective electrodes (ISEs) with ionophore-doped polymeric membranes). The αAstree electronic tongue supplied by AlphaMOS (Toulouse, France) contains cross-selective sensors based on chemically-modified field-effect transistor technology (ChemFET). A second commercial taste sensing system TS-5000Z, produced by Insent (Atsugi-Shi, Japan), consists of a lipidpolymer membrane multichannel electrode, enabling to sense the human taste attributes [18] (whereas ChemFETs working in the αAstree system are not associated to the taste sensations). Ion-selective electrodes based on PVC membranes or chalcogenide glasses were proposed in a laboratory potentiometric electronic tongue system designed for the quantification of tastes, and the differentiation between substances inducing the same taste and estimation of bitter taste of APIs [58]. Recently, an impedimetric electronic tongue was used for the evaluation of taste masking efficacy of Praziquantel encapsulation [59]. Finally, our group elaborated three electronic tongue systems based on various architectures of sensor arrays for the study of pharmaceutical formulations:

- **batch-ET**—system dedicated to batch measurements equipped with sensor array of classical ion-selective electrodes (IS 561, Philips bodies);
- **flow-through-ET**—system dedicated to flow-through measurements equipped with sensor array of miniaturized ion-selective electrodes in customized flow-through cell;
- **FIA-ET (flow injection analysis ET)**—system dedicated to flow injection analysis equipped with sensor array of miniaturized ion-selective electrodes in customized flow-through cell.

**Figure 1:**
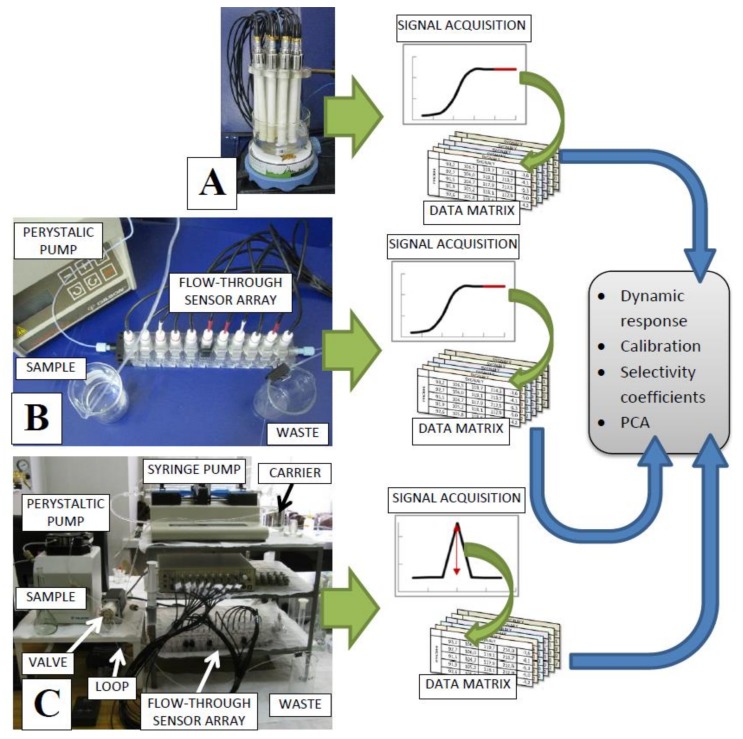
Three developed architectures of electronic tongues: (**A**) Classical set-up, (**B**) flow-through set-up, (**C**) FIA set-up. Reprinted from [60] Copyright 2015, with permission from Elsevier.

Sensor arrays of different architecture provide varied response signals, which are processed using multivariate data analysis methods (Figure 1).

A classical batch set-up (batch-ET) consisted of at most 16 ISEs (the number of channels in the potentiometric multiplexer), which were immersed in studied sample (Figure 1A). Our flow-through-ET comprised a modular flow cell with miniaturized ISEs of classical architecture connected with each other (Figure 1B; detailed description of the structure of modular flow system is a subject of polish patent application [61]). The sample is continuously pumped into the cell, which results in semi-state response signals of each sensor, similar to those obtained in batch-ET (see Figure 1). The constructed flow-through sensor arrays were successfully applied in our previous works for the analysis of foodstuff and environmental samples as well as for the monitoring of biotechnological processes [62,63,64,65]. FIA-ET system comprised the same detector (i.e., modular flow cell with miniaturized ISEs of classical architecture connected with each other), two-position multi-channel valve, and sample injection device with a loop for precise dosing of the injected sample (Figure 1C). In this case, the carrier solution is continuously pumped into the detector cell and the sample is periodically introduced into the carrier stream, forming response signals in the form of peaks [60]. The great advantage of FIA is a large reduction of sample volume needed for the analysis.

The flow operation mode is more advantageous due to the possibility of substantial automatization and shortening of the analytical procedure, leading to the high-throughput analysis of samples [66]. However, it should be emphasized that for the first time our group employed flow modes ETs for pharmaceutical analysis. The electronic tongue dedicated to pharmaceutical analysis consisted of cross-sensitive (CS—cation selective and AS—anion selective) and inorganic cation (Na^+^, K^+^, Ca^2+^)-specific electrodes. Detailed description on preparation of PVC membranes and composition of the internal filling solutions as well as conditioning solutions can be found elsewhere [26,60,62,64]. Three electronic tongues based on various architectures were compared in terms of working parameters and capabilities to recognize taste-masked samples containing ibuprofen [60]. In general, all the systems were characterized by similar performances confirming their suitability for the estimation of the taste masking effect, although the FIA procedure was significantly shorter compared to other approaches. The highest signal repeatability was noticed for the device based on classical electrodes, whereas the FIA-system exhibited the worst repeatable responses (probably due to continuous switching of analyte and carrier solutions during the calibration procedure). The correctness of classification of the pharmaceutical samples (i.e., the ratio of the number of correctly classified samples to the number of all samples) performed by PLS-DA was comparable applying the classical and flow-through systems; however, slightly higher efficiency of the discrimination between pure and modified API was remarked in the case of the FIA-ET system.

In our further studies, sensor arrays comprising cross-sensitive ISEs with only cationic or anionic exchangers as electroactive compounds carbonate/carboxy-selective and amine-selective ISEs were proposed. Performance of an electronic tongue based on classical ISEs (batch-ET) and FIA-ET was compared with two commercially available systems (αAstree and TS-5000Z) and two laboratory prototypes (potentiometric and voltammetric) in independent, interlaboratory studies [57]. The obtained results revealed that laboratory prototypes of potentiometric electronic tongues as well as the αAstree system recognized the studied pharmaceutical formulations in a rather similar manner. However, it was pointed out that only two systems—voltammetric electronic tongue and our batch-ET—were able to differentiate samples containing API from placebo samples, when all samples were simultaneously processed. This can be beneficial for studying taste-masking effects and drug-release kinetic.

Finally, the quantitative analysis of mixtures of APIs was performed using a flow-through electronic tongue [67]. A sensor array composed of eight miniaturized ISEs was connected with the sequential injection analysis (SIA) system providing automated operation and generation of eighty samples of mixtures, containing three APIs in different concentration ranges, thanks to precise dosing and mixing of stock solutions. Such automated system was advantageous due to significantly shorter time of the experiment and seems to be effective for routine quality control of pharmaceutical products.

## 3. Experimental Conditions and Measurements Protocols

**Figure 2:**
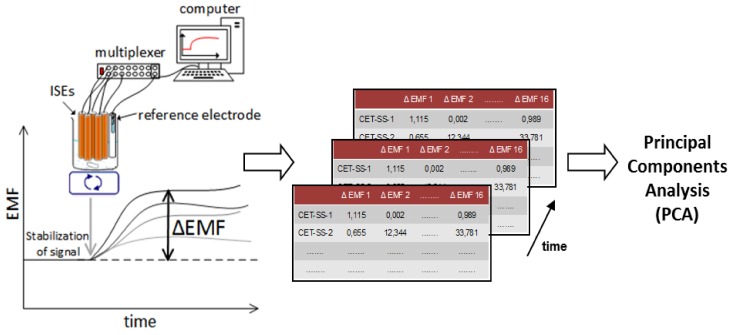
Acquisition and analysis of ET signals. Reprinted from [72] Copyright 2017, with permission from Elsevier.

Conventional dissolution tests of pharmaceutical oral dosage forms performed by recommended apparatus are described in details in Pharmacopeias [68], since various factors such as temperature, pH, medium composition, ionic strength, and hydrodynamics affect this process. On the other hand, studies on pharmaceutical formulations carried out using various electronic tongues described in the literature significantly differ in terms of experimental conditions, although the same factors influence the obtained results. The qualification of electronic tongue systems was performed, but concerned only commercially available devices [69]. One of many factors determining the measurement conditions, which may affect electronic tongues results, has been considered (i.e., the effect of temperature on the αAstree system results was described on the basis of the studies of model solutions and food samples) [70]. Nevertheless, the standardization of the analysis performed by multisensory taste sensing systems is very desirable and should provide detailed protocols of measurements. Therefore, we examined various experimental factors, such as temperature, pH, and composition of the medium, in order to determine their influence on electronic tongue dissolution studies of pharmaceutical formulations. Moreover, the performance of the electronic tongues working in various hydrodynamic conditions was compared testing two systems with different architecture of the sensor array (but similar composition of the ion-selective membranes used in the electrodes) [71]. Minitablets containing valsartan were used as model pharmaceutical samples. A measurement procedure dedicated to dissolution studies of pharmaceutical formulations was developed and applied to all our further studies. The investigated pharmaceutical sample was introduced to the medium after stabilization of the sensors responses, then dissolution/releasing of the API and excipients caused changes of the signals in time–ΔEMF. Subsequently, those changes were processed using pattern recognition techniques; especially PCA was often applied due to the possibility of visualization of obtained chemical images of the samples and their changes over time (Figure 2).

**Figure 3:**
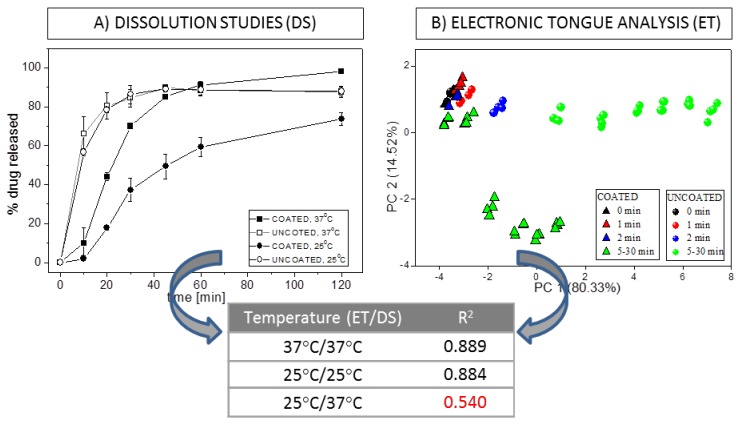
(**A**) Dissolution profiles of valsartan minitablets, (**B**) PCA plot during the release process carried out at 37 °C, and correlation coefficients calculated between the amount of API released during classical dissolution studies conducted at various temperature and distance in PC1–PC2 space. Adapted from [71].

It was evident that the temperature of the medium (37 °C), higher than those commonly used in electronic tongue experiments, improved electronic tongue results. The obtained chemical images of samples were more discernible and correlated better with reference dissolution studies. It must be underlined that very poor correlation of the results was obtained in the most common case (i.e., the measurements carried out at room temperature using the electronic tongue) and reference pharmacopeia dissolution studies performed at 37 °C, while correlation of the results of measurements conducted at the same temperature was significantly higher (see Figure 3). On the basis of our results, we strongly recommend electronic tongue measurements to be performed at temperatures similar to those found in pharmacopeia dissolution studies. Furthermore, the pH and ionic strength of medium was proved to influence electronic tongue results as well. Phosphate buffer pH 6.9 prepared accordingly to European Pharmacopeia should be used to electronic tongue dissolution studies, whereas the second investigated medium—artificial saliva (higher ionic strength, pH 6.8)—is more suitable to taste-masking evaluation. Finally, comparing different architectures of the sensor array, it was observed that flow-through detection mode not only shortened the duration of the experiment but also improved electronic tongue performance. Sensors’ responses of the flow-through electronic tongue were more repeatable and provided more distinguishable chemical images of samples. However, such outcomes might be affected by filtering step, applied during flow-through measurements in order to protect ion-selective membranes from undissolved particles of pharmaceutical formulations.

Two electronic tongues with similar sensor arrays were applied to another dissolution study of modified-release pharmaceutical formulations containing either metamizole sodium or pseudo-ephedrine sulphate [73]. The sensor array of the ET for batch measurements consisted of 16 classical ion-selective electrodes, whereas the sensor array of the FIA-ET included eight miniaturized ISEs. In this case, the flow-through cell was connected to the sequential injection analysis system (FiaLab 3500). The results obtained using FIA-ET were in good accordance with those provided by batch electronic tongue; however, they were slightly difficult to interpret. Similar arrangements of the chemical images of samples were observed in the obtained PCA plots, although in the case of FIA-ET the chemical images of samples were more scattered over the PCA plot (i.e., samples presented a higher variance). This might be a result of the reduced composition of the sensor array and more complicated hydrodynamic conditions during the experiments. Modified-release granules were also investigated at 37 °C employing deionized water and 10^−3^ M HCl as media. Similar results were obtained in both cases.

Additionally, in order to standardize the analysis performed by an electronic tongue, a quick calibration step was introduced every day before the examination of the pharmaceutical samples. The sensitivity of the sensors was tested by measuring the EMFs while increasing the concentration of KNO_3_ in the range 10^−4^−10^−2^ M. Afterwards, ISEs were immersed in stock solution (10^−3^ M NaCl) for stabilization of signals. Such a measurement protocol enabled the verification of proper performance of ISEs and ensured their signals were repeatable [74].

## 4. Selection of Investigated Samples

**Figure 4:**
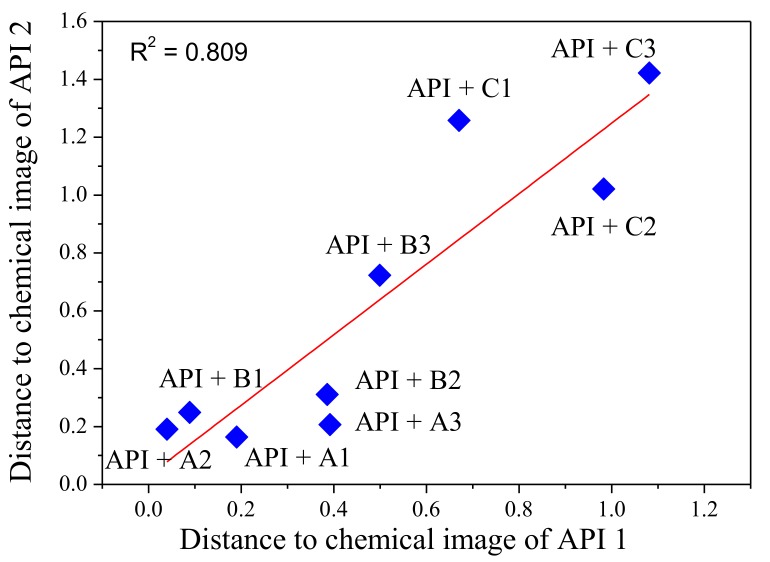
Comparison of relative distances in PC1–PC2 space: Calculated distances for metamizole sodium (API 1) and pseudoephedrine sulphate (API 2) mixtures with three different excipients (EXP-A,-B,-C) at various drug-to-polymer ratios (1,2,3). Reprinted from [74] Copyright 2017, with permission from Elsevier.

Depending on the aim of pharmaceutical analysis, the samples may be distributed into different numbers of categories. In the most common case, three types of samples are examined by electronic tongue: taste-masked/modified-release pharmaceutical formulation (i.e., API with excipients), pure API, and placebo (i.e., pharmaceutical formulation without API, as a control). The recorded signals of the sensors are processed using multivariate techniques, mostly the principal component analysis. The efficiency of taste masking is mainly assessed on the basis of the distances (generally Euclidean distance is calculated) between points corresponding to pharmaceutical formulations, pure bitter API, and placebo on the PCA plot. The farther the taste-masked formulation is placed from API and nearer to the placebo, the more efficient taste-masking is suggested. The classification results for different pharmaceutical samples (e.g., modified-release formulations) are interpreted in a similar manner. Various other methods like PLS-DA (partial least squares-discriminant analysis), ANN, or SVM are employed to construct models predicting bitterness, concentration of API in examined samples, as well as to correlate the results obtained by electronic tongue and reference method, like sensory panel or dissolution studies. These supervised classification techniques are based on learning by examples (i.e., a model is developed by learning to assign a sample to an unknown class using a training set of features patterns of samples with known class membership). Sensor array responses are affected by API as well as excipients present in solution. Therefore, the obtained differences in chemical images of samples might be a result of modification of the API release rate by appropriate excipient (e.g., lower accessibility of API at the first stage of releasing process caused by encapsulation) and/or simultaneous sensing of various substances (API and excipients)—so-called “mixture effect”. Such phenomenon significantly hinders the interpretation of electronic tongue results and might lead to incorrect conclusions. In order to study the “mixture effect” and describe the influence of excipient on the chemical images of APIs, physical mixtures of exemplary APIs with different ionic properties (metamizole sodium and pseudoephedrine sulfate) with common polymer excipients used in pharmaceutical products were tested [74]. After mixture dissolution, each sample contained the same amount of API (10^−3^ M) and different amounts of excipient at three various drug-to-polymer ratios (corresponding to ratios found in commercially available pharmaceutical products). Batch-ET consisted of 16 ISEs of various cross-sensitivity according to different electroactive additives used in this study. The obtained results indicated the influence of excipients on sensor array responses measured in API solutions (in other words different discrimination abilities of the sensor array). However, high correlation of the distances on the PCA plot calculated for the samples with different APIs proved that the excipients alter the chemical image of API regardless of the structure of API, though it is quantitatively related with the sensor sensitivity (Figure 4). The pronounced influence of the excipient on the chemical images of API might be a result of its higher concentration than API in the solution, despite favorable electrode selectivity towards API. The scale of this alteration depends on a whole sensor array capability to detect API and excipients, as well as individual sensors sensitivity towards particular ingredients of the samples. Our results indicated that not only pharmaceutical formulations, but also physical mixtures with the same composition, should be considered during electronic tongue experiments in order to elucidate the causes of changes of chemical images of samples (effective taste-masking/modified release or “mixture effect”).

In our further studies we continued to investigate the impact of selected dissolution-modifying excipients on the release process as well as on the outcomes of the sensor array. The dissolution of modified-release three types of the granules containing either metamizole sodium or pseudoephedrine sulfate and one of two cellulose derivatives (carmellose sodium or hypromellose), as well as mixtures of the same components, were examined using batch-ET and FIA-ET. Wet granulation process was used for granules preparation, while mixtures were obtained using laboratory scale mixer. API and placebo samples were also analyzed [73]. It was noticed that the release process of API was affected by the presence of polymers in a different degree. Mixture and granules containing metamizole sodium and carmellose sodium were similarly sensed by the electronic tongue (i.e., similar chemical images were obtained), which means that the changes of chemical images of samples over time might be a result of either simultaneous detection of API and excipients or efficient modified release of API. On the other hand, completely different chemical images of granules and mixtures of the same composition were noticed for other pharmaceutical formulations (pseudoephedrine sulphate and hypromellose; metamizole sodium and hypromellose). Therefore, the influence of granulation process on chemical images was proved. In conclusion, the examination of dosage forms as well as physical mixtures of API and excipient are needed for proper analysis of the efficiency of release modification. Moreover, a reliable elucidation of the results provided by the electronic tongue should also take into account the selectivity and sensitivity of the sensors towards APIs/excipients.

## 5. Analysis of the Signals of Sensor Arrays

The analytical tasks, which the electronic tongue systems attempt to solve are qualitative or quantitative. The qualitative approach includes mainly the recognition and/or classification of samples on the basis of their distinctive properties, allowing to identify, for instance, organoleptic characteristics or geographical origin. On the other hand, quantitative applications concern the simultaneous calibration of multiple analytes or the prediction of characteristic properties and/or sensorial attributes. Since electronic tongue measurements provide multidimensional data, the multivariate approach offered by chemometric tools is preferred [24]. Processing of the sensor array signals includes extraction of the valuable features, data pre-treatment, and, finally, development of a classification/prediction model. Each step should be performed with caution due to possible errors strongly affecting the obtained results. Steady-state (signals recorded during last seconds of measurements) or dynamic responses (signals recorded during first seconds of measurement or whole recorded signals) of the sensor array are mainly processed by pattern recognition techniques. PCA, wavelet transformation (WT), and genetic algorithms (GAs) can be employed to extract valuable features from recorded signals as well. Afterwards, multivariate data analysis is performed using various chemometric tools. In most cases, PCA is the prime choice to get the first impression on the complexity of data and to visualize the chemical images of samples. The most popular classifiers are PLS-DA, K-nearest neighbors (KNN), and SVM, while PLS, PCR (principal component regression), and ANN are generally used for quantitative tasks (ANN is a common classifier as well). There is no single appropriate method to process sensor array data, providing reliable results and low levels of error, which can be applied to every classification task. In the literature, the proposed approaches are significantly varied in terms of employed chemometric techniques as well as feature extraction methods.

Since data processing is an essential step of electronic tongue analysis, we focused on the appropriate choice of pattern recognition techniques for the pharmaceutical studies as well. A flow-through-ET was applied to the quantitative analysis of mixtures containing three APIs in different concentration ranges [67]. Steady-state responses as well as dynamic responses of the sensor array were processed by PLS, three-way PLS, and ANNs in order to enhance the prediction abilities of the developed system. Various variants of training matrices were considered, although the application of PLS and three-way PLS led to the proper prediction of the concentration of two APIs out of three. The use of non-linear classifier with the extraction of dynamic components of the transient response by the Wavelet transform, and proper pruning and training of an ANN with the selected coefficients, allowed the simultaneous determination of the three APIs. Therefore, it was evident that the prediction abilities of electronic tongue systems are strongly influenced by the used chemometric methods. Moreover, appropriate methods of feature extraction and reduction of insignificant data are also advantageous. The role of the chosen pattern recognition technique was also investigated during the classification of pharmaceutical taste-masked samples using batch-ET equipped with 16 ISEs. The most frequently used techniques for the processing of sensor arrays signals were compared: PCA, PLS-DA, three-way PLS-DA, SIMCA (soft independent modelling of class analogy), PCR-DA, SVM, and KNN, as well as fusion of PCA and back propagation neural networks (BPNN). Many models with various typical parameters were developed for the proper optimization of each classifier. Moreover, the influence of the feature extraction method on the classification abilities of the electronic tongue was determined by processing different data matrices. The obtained results indicated that the adjustment of typical parameters of the classifier plays a key role in the data processing and might remarkably improve the results of electronic tongue analysis. Generally, the investigated pattern recognition techniques exhibited considerably varied classification abilities. The highest number of samples were correctly classified using SVM as well as fusion of PCA and BPNN (whereas PCR-DA and KNN correctly assigned the least samples), considering the steady-state responses of the sensor array (i.e., the most popular method of data feature extraction). Depending on the used pattern recognition technique, different feature extraction methods may be advantageous. Therefore, we proposed some recommendations for the methodology of electronic tongue analysis e.g., the application of steady-state responses for simpler classifiers (KNN) and dynamic responses for other classifiers (PLS-DA, SIMCA, ANN), and the use of both linear as well as non-linear classifiers for data analysis of pharmaceutical samples [75]. Our findings might be helpful for all researchers working on processing sensor array responses, with particular emphasis on investigation of the taste-masking efficiency carried out using laboratory and commercial electronic tongues.

## 6. Reference Methods

Despite almost 30 years of studies on electronic tongue systems, reference methods are still required to verify the results obtained using multisensory arrays. So far, artificial sensing systems are considered as supplementary devices to conventional analytical methods. The evaluation of organoleptic properties of pharmaceutical samples, including flavor, is performed by human taste panel. This method involves healthy volunteers assessing taste on the basis of predefined numerical scale or score. However, those tests arouse ethical and toxicological concerns especially when drug of interest is still on early stage of development. The difficulty of this test lies in the subjectivity of human taste sensation, because it depends on nationality, sex, eating habits, etc. Nevertheless, such a test is still the most reliable method for the assessment of taste-masking efficiency; therefore, some authors correlated the results obtained using electronic tongue systems with in vivo analysis [47,58].

**Figure 5:**
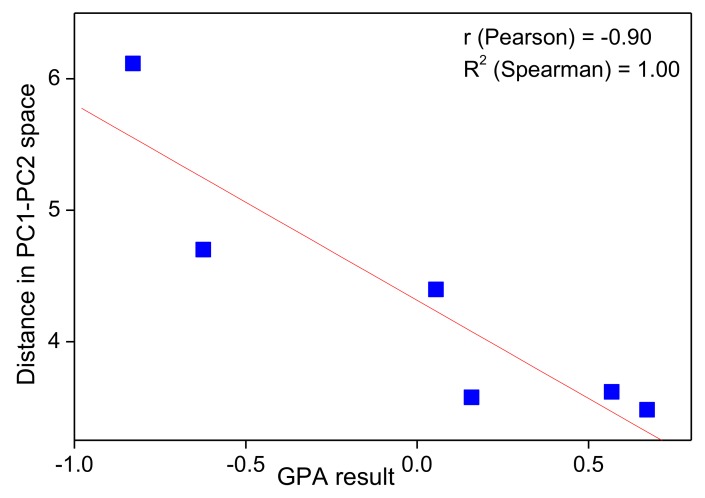
Correlation between Euclidean distance of chemical images of investigated formulations to API on PCA plot and human taste panel results (GPA scores). Reprinted from [72] Copyright 2017, with permission from Elsevier.

In our studies, an electronic tongue consisting of 16 classical ISEs (batch-ET) was employed for taste evaluation of cetirizine-based microspheres [72]. The results were correlated with human taste panel assessment. The responses of the sensor array, recorded in medium with microspheres containing cetirizine dihydrochloride and polymer coating agent (with different drug-to-polymer ratio), were processed by PCA. For a deeper understanding of human panel results, “compromise scores” were calculated using generalized procrustes analysis (GPA). GPA is one of the most commonly applied approaches to cope with sensory profiling data (sensory panelists’ responses). It calculates a weighting factor which compensates for differences in the use of scale among individual testers and therefore, evidencing statistically significant distinctions in responses of panelists [76]. Electronic tongues results were found to be consistent with the reference studies. High correlation (R^2^ Spearman = 1.00) was obtained between results of GPA and calculated distances between chemical images of taste-masked microspheres and API on PCA plot (Figure 5). In other words, the higher bitterness was assigned to the microsphere sample, the closer to API its chemical image was located on the PCA plot.

The alternative method, often used as a reference to electronic tongue investigations, involves conventional drug dissolution studies. The amount of API released from solid dosage forms to medium simulating physiological fluids is determined usually by HPLC or UV–Vis spectroscopy. During the studies on cetirizine-based microspheres, conventional drug dissolution tests were performed as well [72]. Partial inconsistency of drug dissolution results and human taste panel evaluation was unexpectedly observed. This might be a result of various hydrodynamic and temperature conditions as well as time scales of both experiments. Therefore, it can be seen that these two routinely used procedures supply different information on examined samples and are not equivalent but rather complementary (the position of chemical image on PCA plot could be related to the results of dissolution studies). Once again, it was proved that the developed device provides information on both the amount of the released API as well as excipients present in the samples.

Drug dissolution tests were also used as a reference method in our studies on modified-release pharmaceutical formulations conducted with the electronic tongue [71,73]. High correlation (R^2^ = 0.889) of electronic tongue results with reference dissolution studies was obtained testing minitablets containing valsartan (although it should be emphasized that similar conditions of both experiments were essential for good accordance of the outcomes). During the examination of granules performed by the electronic tongue, partial inconsistency was remarked between the developed multisensory system and the reference studies. It might be related with the specific characteristics of the ISEs forming the sensor arrays (mainly their limited selectivity and thus simultaneous sensing of API and excipient as well as varied sensitivity towards API and excipient). Therefore, the ET results should be interpreted and correlated with reference dissolution studies with caution, taking into account the characteristics of particular sensors and the whole sensor array.

## 7. Conclusions

Electronic tongue systems equipped with cross-sensitive potentiometric electrodes are gaining special interest in pharmaceutical analysis due to the possibility of various applications: studying taste masking effects, investigating the release kinetics of pharmaceuticals, and developing new formulations. As a novel approach their results must be compared to conventionally applied methods, among which human taste panel and dissolution tests are gold standards provided by Pharmacopeias. Many studies already proved the complementarity between electronic tongue and classical analysis. However, it must be emphasized that, in contrast to conventional methods, there are no pharmacopeia regulations on electronic tongue applications for pharmaceutical analysis, considering many issues affecting the results obtained by such systems: sensor performance, sensor array composition, measurement conditions, and data analysis. Moreover, the literature researches show that commercial versions of electronic tongues are mainly used for pharmaceutical analysis. We believe that at this stage of development of electronic tongue systems, still much work should be done by sensor specialists to present protocols applicable for standardization of electronic tongue results.

This work presented our experience related to the development of electronic tongue systems dedicated to pharmaceutical analysis. We underlined a need for studying sensor performance, especially the ability to sense both API and excipients. The composition of the sensor array and repeatability of signals are also crucial and should be considered to avoid non reliability of the results. Measurement conditions, such as temperature during drug release, hydrodynamic parameters (batch/flow-through/FIA detection mode), and medium composition, all have strong impact on the results obtained, and, when chosen improperly, may lead to inconsistency with reference studies. Even the extraction of information from sensor signals (steady-state responses vs dynamic signals) and data analysis can be improved for better clustering of samples and thus higher classification rates.

Finally, it should be stressed that, in most works presented in the literature, all above issues (i.e., the composition and architecture of the sensor array, performance of the sensors, experimental conditions, selection of the investigation samples, method of feature extraction and data processing) are overlooked; however, their detailed analysis could improve the reliability and interpretability of the results and at the same time could strengthen the correlation with standardized reference studies. This overview aims at filling this gap by critical discussion of all these aspects step-by-step, and may be helpful for better understanding of electronic tongue systems development as well as for providing strict testing protocols and specification limits of these devices.
